# Supplementary material for: Origin, trophic transfer and recycling of particulate organic matter in two upwelling bays of Humboldt Current System: Insights from compound-specific isotopic compositions of amino acids
Source: PLoS One. 2025 May 14;20(5):e0306674. doi: 10.1371/journal.pone.0306674 (PMC12077699; doi:10.1371/journal.pone.0306674)
Supplement: S1 Data — S1 Fig. Vertical profiles of Chlorophyl-a, and NH4+ contents in MB (A and C) and AB (B and D). S2 Fig. Vertical profiles of NO2-, and NO3- contents in MB (A and C) and AB (B and D). S3 Fig. Vertical profiles of PO43-, and TOC contents in MB (A and C) and AB (B and D). S4 Fig. (A) A box plot accompanied by jitter points illustrates the dissolved oxygen inventories in the water columns of MB and AB. Additionally, vertical profiles of water column stratification, represented by the Brünt-Väisälä frequency, are presented for both MB (A) and AB (B). S5 Fig. Vertical profiles of mean values for (A) δ15N Phe-normalized Tr-AA, and (B) Sr-AA in the water columns of MB and BA. Data are presented as mean ± standard deviation values. S6 Fig. Box and jitter plots of (A) δ15N and (B) δ13C of THAA in suspended POM from MB and AB. S7 Fig. Bayesian stable isotope mixing model (MixSIAR) applied to δ15N end-members in POM samples from two depths in Mejillones and Antofagasta Bays. The top panel shows results from Mejillones Bay at two depths: 5–20 m (A-B) and 35–45 m (C-D). Panels A and C display the range of variability for the end-members, along with the POM data at their respective depths, and density plots illustrating the prior and posterior distributions for each source. Panels B and D depict the relationships (distributions and correlations) between the different sources within the mixture. The bottom panel shows the same distributions for Antofagasta Bay. S8 Fig. Biplot data on phenylalanine (Phe)-normalized threonine (Thr) and alanine (Ala) δ15N values for end-members (microbially degraded organic matter, MDOM; fecal pellets, FP; phytoplankton; and zooplankton) as well as suspended and sinking POM from MB and AB are presented as means ± standard deviations. Suspended and sinking POM data are from this study. Other data are from published studies, detailed in Table S1. S1 Table. Phe-normalized δ15N values of Thr and Ala used in S7 Fig., with metadata. S2 Table. Full data on δ1 [file pone.0306674.s001.pdf]

Supporting Information

Srain, B.M, Flores, E, Valdés, J., Camaño, A

S1 Figure. Vertical profiles of Chlorophyl-a, and NH_4^+ contents in MB (A and C) and AB (B and D).

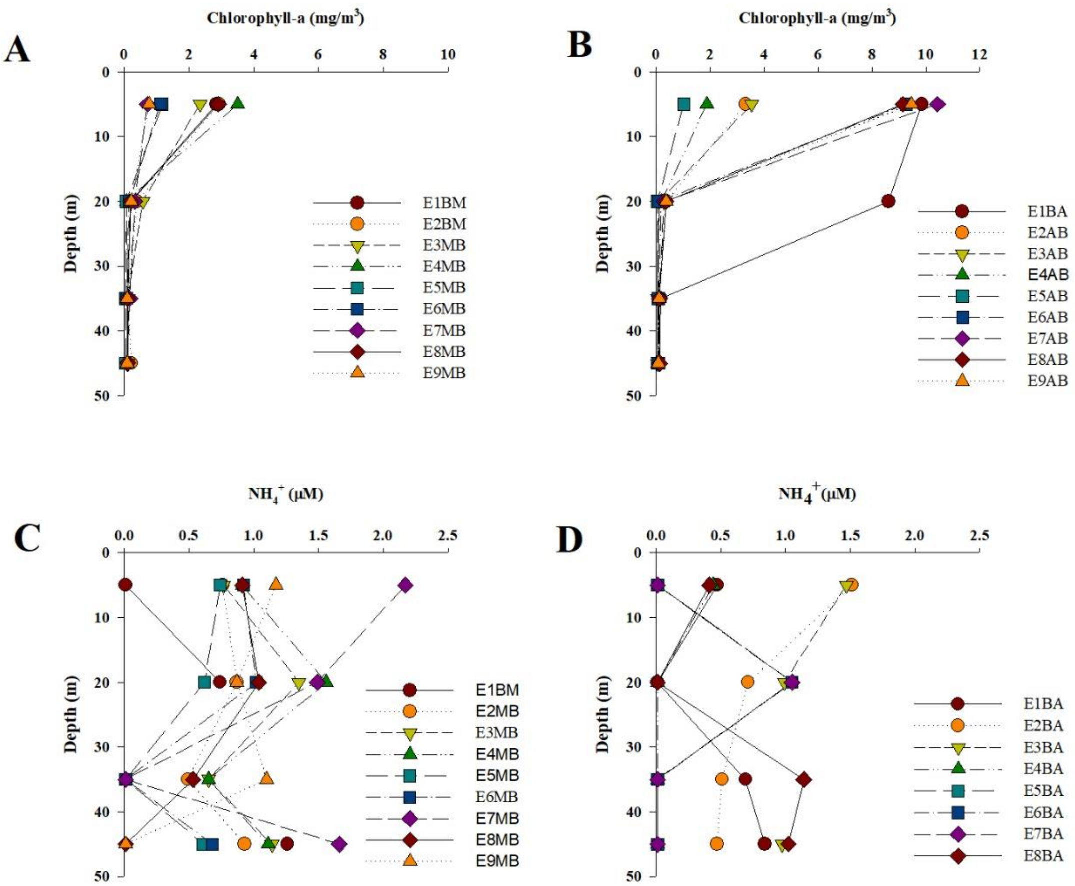

S2 Figure. Vertical profiles of NO_2^-, and NO_3^- contents in MB (A and C) and AB (B and D).

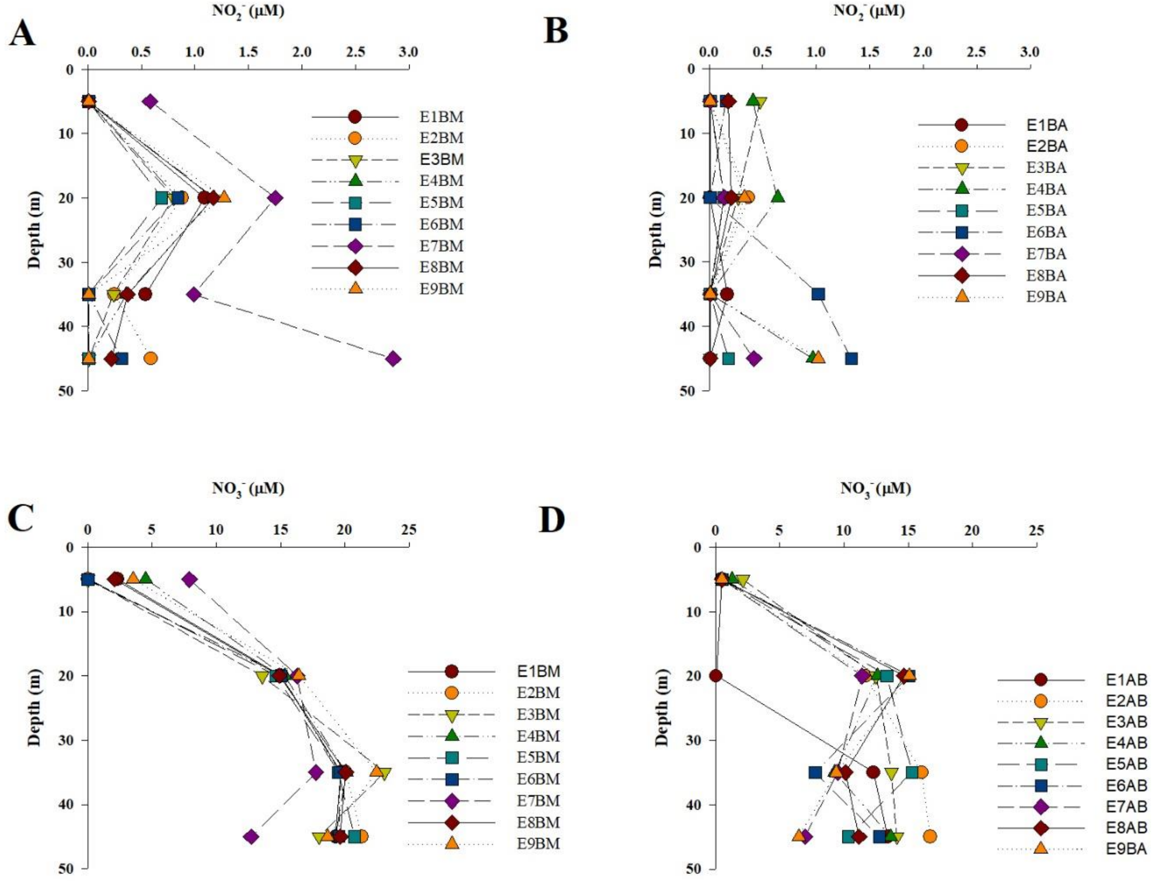

S3 Figure. Vertical profiles of PO_4^{3-}, and TOC contents in MB (A and C) and AB (B and D).

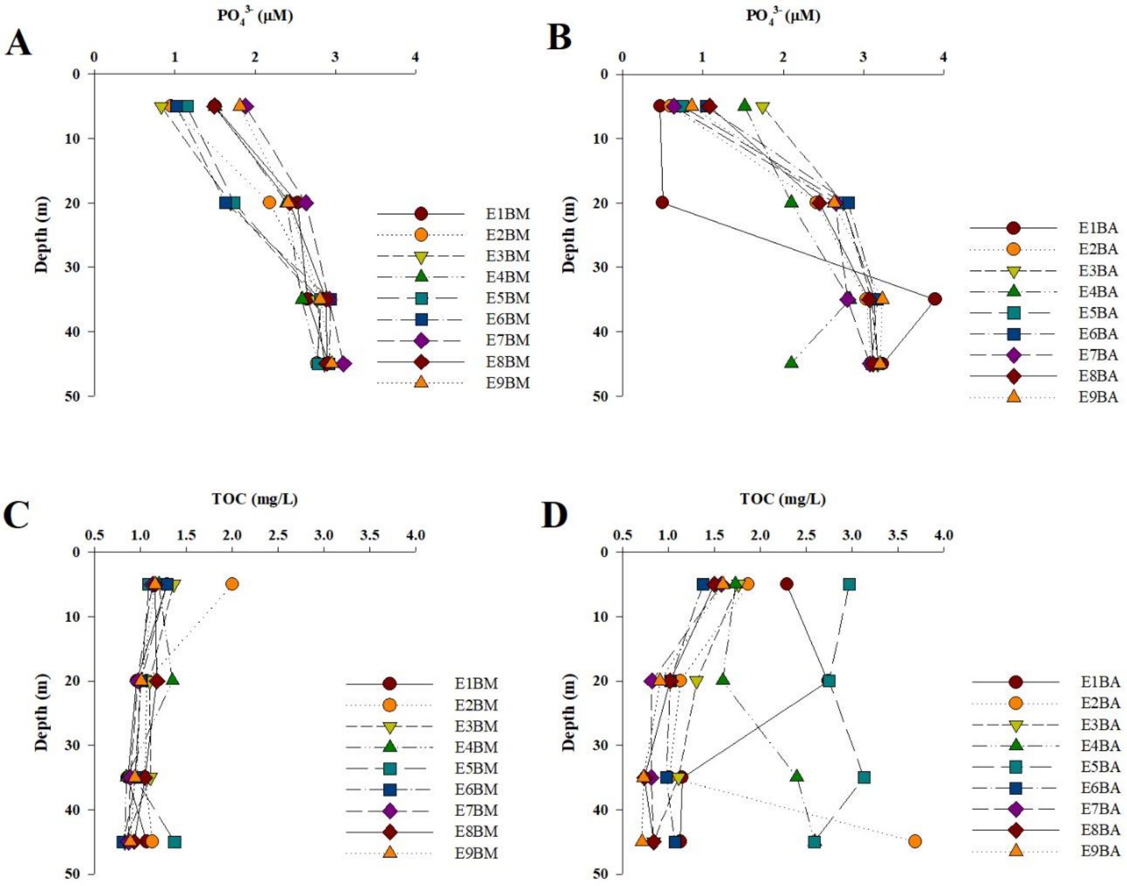

S4 Figure. (A) A box plot accompanied by jitter points illustrates the dissolved oxygen inventories in the water columns of MB and AB. Additionally, vertical profiles of water column stratification, represented by the Brünt-Väisälä frequency, are presented for both MB (A) and AB (B).

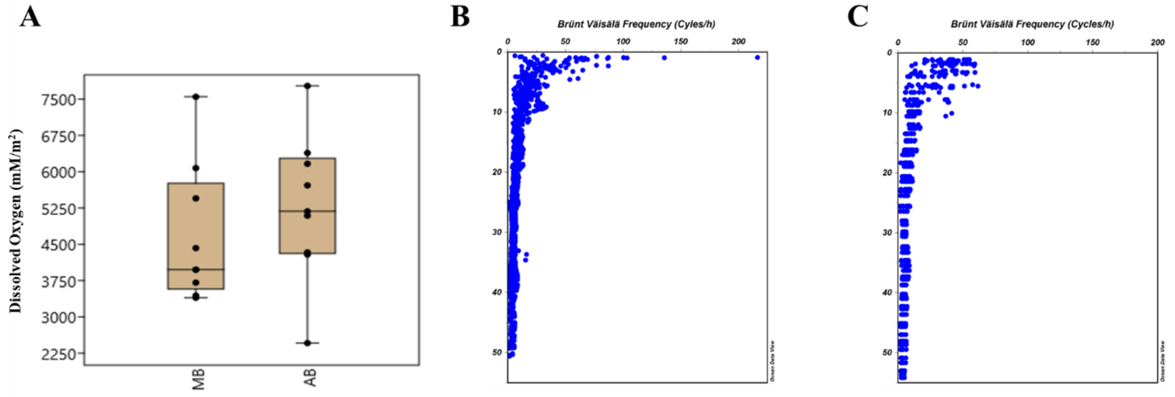

S5 Figure. Vertical profiles of mean values for (A) δ^{15}N Phe-normalized Tr-AA, and (B) Sr-AA in the water columns of MB and BA. Data are presented as mean ± standard deviation values.

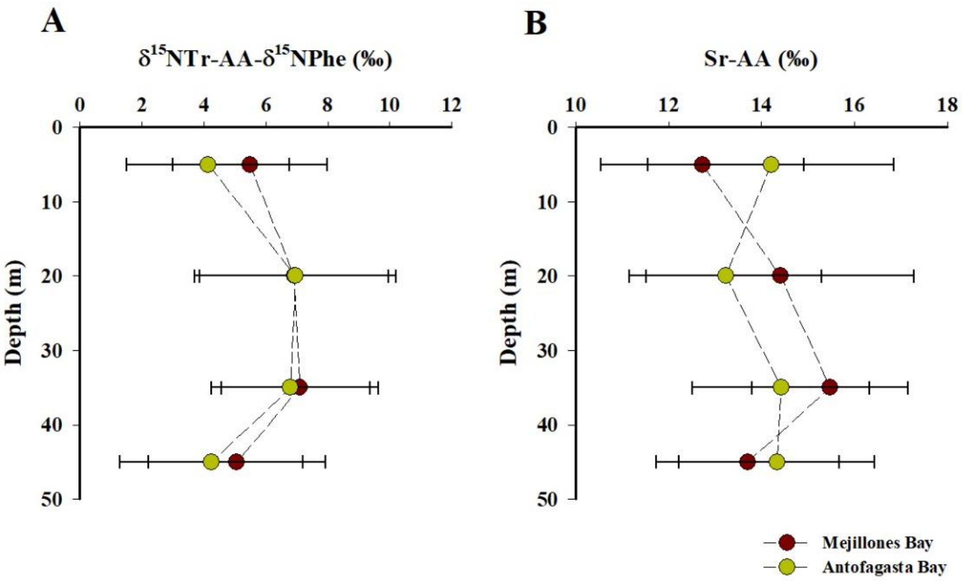

S6 figure. Box and jitter plots of (A) $\delta^{15}N$ and (B) $\delta^{13}C$ of THAA in suspended POM from MB and AB.

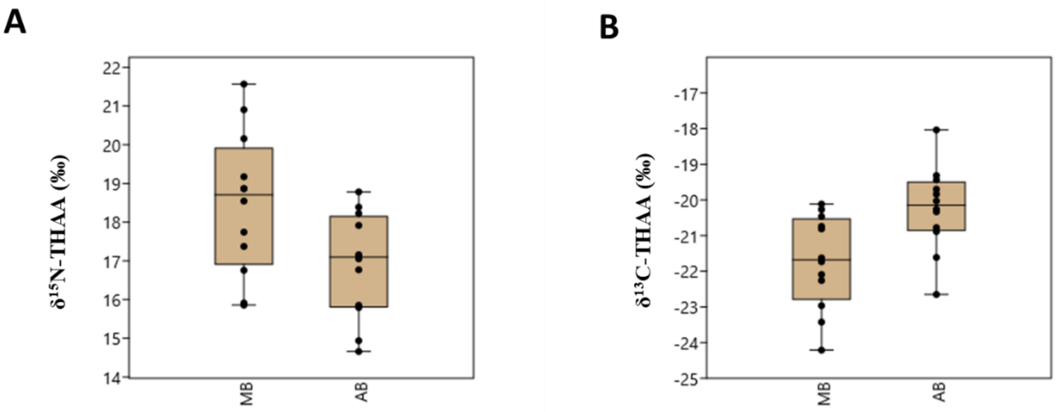

S7 Figure. Bayesian stable isotope mixing model (MixSIAR) applied to $\delta^{15}N$ end-members in POM samples from two depths in Mejillones and Antofagasta Bays. The top panel shows results from Mejillones Bay at two depths: 5-20 m (A-B) and 35-45 m (C-D). Panels A and C display the range of variability for the end-members, along with the POM data at their respective depths, and density plots illustrating the prior and posterior distributions for each source. Panels B and D depict the relationships (distributions and correlations) between the different sources within the mixture. The bottom panel shows the same distributions for Antofagasta Bay.

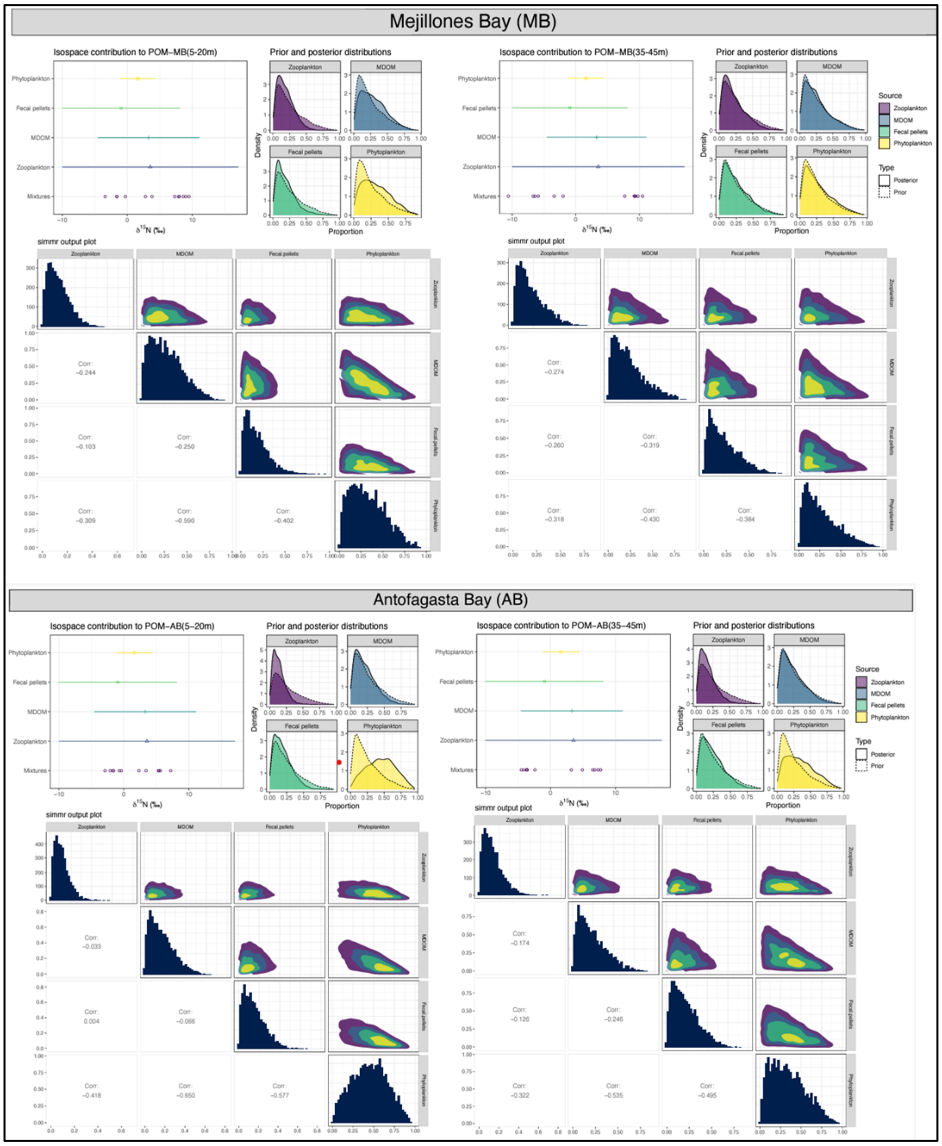

S8 Figure. Biplot data on phenylalanine (Phe)-normalized threonine (Thr) and alanine (Ala) δ15N values for end-members (microbially degraded organic matter, MDOM; fecal pellets, FP; phytoplankton; and zooplankton) as well as suspended and sinking POM from MB and AB are presented as means ± standard deviations. Suspended and sinking POM data are from this study. Other data are from published studies, detailed in Table S1.

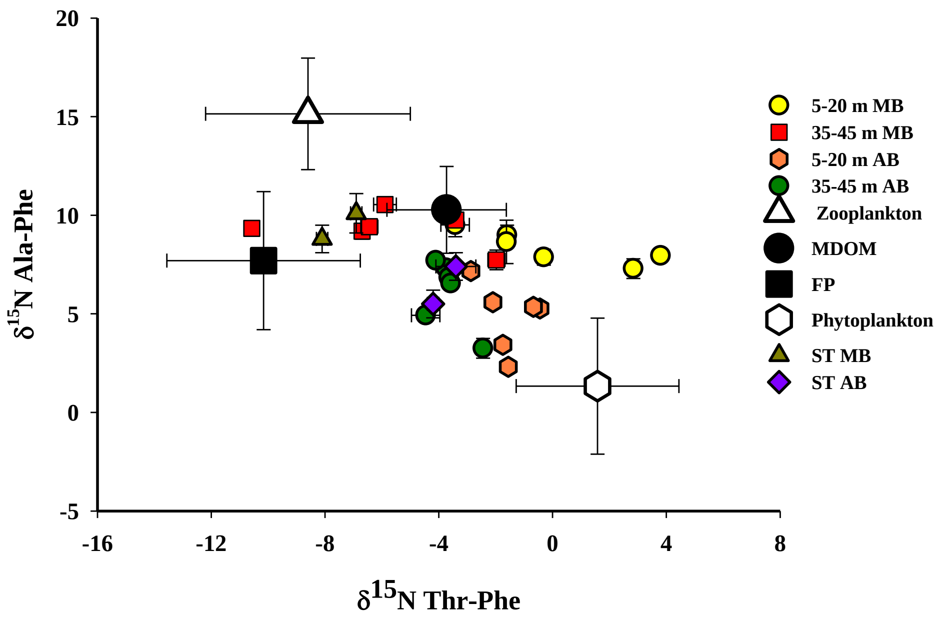

S1 Table. Phe-normalized $\delta^{15}N$ values of Thr and Ala used in S7 Fig., with metadata.

End member	Source	Collection location	$\delta^{15}N$Thr-Phe (‰)	$\delta^{15}N$Ala-Phe (‰)
Zooplankton biomass	Hannides et al., (2020)	Station Aloha	-7.2	12.7
			-9.3	14.0
			-8.7	16.5
			-10.9	18.1
	Hannides et al., (2009)		-11.0	17.9
			-8.6	19.0
			-7.9	20.1
			-10.0	18.7
			-7.4	14.6
			-6.8	14.1
			-7.6	14.0
			-9.8	14.4
			-8.2	15.8
			-12.1	12.9
			-9.4	15.1
			-13.6	16.5
			-8.0	14.1
			-10.0	14.3
			-9.4	17.3
			-11.3	15.7
			-10.0	15.5
			-8.6	16.8
	Hannides et al., (2013)		-7.0	11.5
			-7.2	14.3
			-8.4	13.9
			-11.1	16.0
			-9.0	10.3
			-8.95	13.3
			-9.1	14.8
	McClelland & Montoya (2003)	Tropical North Atlantic	-2.8	11.4
			-0.9	10.9
			-4.4	11.2
			-7.2	11.2
			-4.6	10.7
	Romero-Romero et al., (2020)	Equatorial Pacific	-5.4	18.8
			-10.3	17.5
			-10.4	19.3
			-4.5	19.6
			3.5	16.8
			-2.4	11.1
			-14.8	19.8
			-11.8	19.3
			-4.7	16.5
			-13.6	18.7
			-6.8	14.2
			-4.5	13.9
			-3.4	19.9
			-7.7	19.4
	Doherty et al., (2021)	Southern Ocean	-13.1	11.3
		NE Pacific	-15.8	15.3
			-9.9	12.9
		Sargasso Sea	-9.1	12.3
			-12.2	12.5
			-12.7	14.0
			-15.4	12.1

Continuation of S1 Table

End member	Source	Collection location	$\delta^{15}N_{Thr-Phe}$ (‰)	$\delta^{15}N_{Ala-Phe}$ (‰)
Phytoplankton	McCarthy et al., (2013)	Culture	1.0	3.7
			-0.2	6.4
			1.4	2.9
			-0.2	-2.1
			-1.3	-0.5
			6.3	1.9
			-4.5	-3.5
			1.7	-2.3
			2.1	-2.2
			4.4	3.6
			5.3	7.7
	McClelland & Montoya (2013)		3	0.4
Fecal pellets	Doherty et al., (2021)	Southern Ocean	-5.9	9.1
		NE Pacific	-11.1	11.5
			-6.8	1.3
		Sargasso Sea	-10.5	3.6
			-8	9.9
			-12.1	7.8
			-16.7	10.7
MDOM	Yamaguchi & McCarthy (2018)	North Pacific Subtropical Gyre	-6,1	8.6
			-5.5	8.4
			-2.0	10.2
			-1.3	13.9
Suspended - POM	This study	Mejillones Bay, (5-20 m depth)	3.8	8.0
			-1.6	9.0
			2.8	7.3
			-3.4	9.5
			-0.3	7.9
			-1.6	8.6
		Mejillones Bay, (35-45 m depth)	-5.9	10.5
			-6.7	9.2
			-6.4	9.4
			-10.6	9.3
			-3.4	9.8
			-2.0	7.7
		Antofagasta Bay, (5-20 m depth)	-0.4	5.3
			-1.7	3.4
			-0.7	5.4
			-2.9	7.2
			-1.6	2.3
			-2.1	5.6
		Antofagasta Bay, (35-45 m depth)	-2.4	3.2
			-4.5	4.9
			-3.7	7.3
			-3.6	6.8
			-4.1	7.7
			-3.6	6.5
Sinking-POM		Mejillones Bay (Sediment trap)	-8.1	8.8
			-6.9	10.1
		Antofagasta Bay (Sediment trap)	-3.4	7.4
			-4.2	5.5

S2 Table. Full data on $\delta^{15}N$ amino acids in suspended POM collected from MB and AB.

Mejillones Bay								
	**E2MB 5M**		**E2MB 20M**		**E2MB 35M**		**E2MB 45M**	
**AA**	**Mean (‰)**	**SD (‰)**	**Mean (‰)**	**SD (‰)**	**Mean (‰)**	**SD (‰)**	**Mean (‰)**	**SD (‰)**
**Ala**	20.6	0.3	22.3	0.8	25.6	0.5	23.6	0.6
**Gly**	11.2	0.1	11.0	0.3	12.0	0.2	9.7	0.1
**Thr**	16.4	0.2	11.7	0.2	9.1	0.2	7.7	0,1
**Ser**	10.3	0.4	10.8	0.4	12.7	0.4	10,7	0.3
**Val**	20.8	0.0	21.1	0.3	23.9	0.4	21.8	0.5
**Leu**	13.7	0.1	15.2	0.1	17.9	0.2	14.7	0.3
**Ile**	15.1	0.5	16.5	0.3	18.9	0.5	16.5	0.5
**Pro**	17.3	0.3	17.4	0.2	21.3	0.1	20.1	0,3
**Asp**	18.1	0.2	16.7	0.1	20.6	0.3	17.4	0.2
**Glu**	20.5	0.3	20.8	0.5	24.4	0.0	21.3	0.3
**Phe**	12.6	0.2	13.3	0,3	15.0	0.6	14.4	0,2
**Tyr**	12.7	0.6			15.8	0.9		
**Lys**	15.8	0.2	8.9	0.4	14.6	0.5	12.1	0.5
	**E5MB 5M**		**E5MB 20M**		**E5MB 35M**		**E5MB 45M**	
**AA**	**Mean (‰)**	**SD (‰)**	**Mean (‰)**	**SD (‰)**	**Mean (‰)**	**SD (‰)**	**Mean (‰)**	**SD (‰)**
**Ala**	19.8	0.9	23.9	0.1	25.0	0.3	25.2	0.4
**Gly**	11.2	0.7	11.5	0.3	13.6	0.9	13.6	0.8
**Thr**	15.3	0.3	10.9	0.3	9.2	0.2	5.3	0.3
**Ser**	9.7	0.3	11.7	0.3	13.1	0.3	12.1	0.2
**Val**	21.5	0.4	24.1	0.4	25.7	0.5	23.7	0.2
**Leu**	13.4	0.3	17.3	0.3	18.8	0.1	17.8	0.2
**Ile**	15.6	0.5	19.1	0.1	20.5	0.6	19.6	0.4
**Pro**	18.3	0.2	21.8	0.2	22.6	0.4	21.7	0.1
**Asp**	18.1	0.1	20.1	0.2	20.7	0.3	19.2	0.1
**Glu**	20.9	0.7	24.3	0.1	26.3	0.2	23.7	0.2
**Phe**	12.5	0.4	14.4	0.7	15.6	0.4	15.9	0.2
**Tyr**	13.2	0.2	15.7	0.7	17.5	0.9	17.3	0.3
**Lys**	10.2	0.3	15.6	0.2	15.3	0.3	13.5	0.1
	**E7MB 5M**		**E7MB 20M**		**E7MB 35M**		**E7MB 45 M**	
**AA**	**Mean (‰)**	**SD (‰)**	**Mean (‰)**	**SD (‰)**	**Mean (‰)**	**SD (‰)**	**Mean (‰)**	**SD (‰)**
**Ala**	21.3	0.1	25.8	0.3	25.8	0.6	22.8	0.1
**Gly**	9.5	0.1	14.3	0.5	15.1	0.2	13.0	0.2
**Thr**	13.1	0.2	15.5	0.0	12.6	0.3	13.1	0.2
**Ser**	10.5	0.2	14.5	0.1	14.0	0.3	11.7	0.4
**Val**	21.9	0.3	25.8	0.2	25.1	0.4	22.1	0.6
**Leu**	14.3	0.1	18.8	0.1	19.9	0.2	14.7	0.4
**Ile**	17.1	0.2	21.5	0.3	21.5	0.6	16.8	0.2
**Pro**	19.9	0.2	22.7	0.5	22.8	0.4	20.3	0.0
**Asp**	19.0	0.1	23.1	0.2	22.5	0.3	18.4	0.1
**Glu**	22.1	0.1	27.2	0.1	25.8	0.4	22.7	0.2
**Phe**	13.4	0.3	17.1	0.3	16.0	0.3	15.1	0.5
**Lys**	15.9	0.3	17.7	1.3	18.2	0.8		

Continuation of S2 Table

Antofagasta Bay								
	E2AB 5M		E2AB 20M		E2AB 35M		E2AB 45 M	
AA	Mean (‰)	SD (‰)	Mean (‰)	SD (‰)	Mean (‰)	SD (‰)	Mean (‰)	SD (‰)
Ala	18.3	0.3	17.3	0.4	18.4	0.7	21.1	0.5
Gly	10.0	0.3	10.4	0.4	11.3	0.9	11.9	0.3
Thr	12.6	0.2	12.2	0.3	12.7	0.3	11.7	0.3
Ser	9.1	0.1	9.8	0.3	9.9	0.4	10.7	0.1
Val	17.8	0.1	16.9	0.2	20.2	0.5	20.6	0.3
Leu	12.8	0.2	12.1	0.3	13.6	0.5	14.7	0.1
Ile	13.6	0.2	13.5	0.3	14.4	0.4	16.0	0.2
Nle	15.1	0.2	15.3	0.1	15.4	0.1	15.4	0.0
Pro	17.5	0.5	18.1	0.3	18.2	0.1	20.2	0.3
Asp	15.8	0.2	17.1	0.2	16.9	0.2	18.7	0.1
Glu	16.9	0.1	18.1	0.1	18.9	0.3	20.9	0.6
Phe	13.0	0.1	13.9	0.5	15.1	0.1	16.2	0.8
Tyr	15.8	0.1			14.8	1.1	16.7	0.4
Lys	16.9	0.4	13.9	0.3	14.8	0.1	14.6	0.4
	E5AB 5M		E5AB 20M		E5AB 35M		E5AB 45 M	
AA	Mean (‰)	SD (‰)	Mean (‰)	SD (‰)	Mean (‰)	SD (‰)	Mean (‰)	SD (‰)
Ala	20.1	0.2	22.2	0.4	23.9	0.3	23.1	0.9
Gly	10.3	0.5	12.4	0.3	13.7	0.3	12.9	0.6
Thr	14.1	0.3	12.1	0.4	12.8	0.4	12.6	0.5
Ser	10.3	0.3	11.2	0.5	12.8	0.3	11.9	0.6
Val	21.1	0.3	22.2	0.5	23,0	0.4	22.0	0.2
Leu	14.5	0.2	15.8	0.2	16.2	0.2	15.7	0.2
Ile	15.5	0.2	17,0	0.2	18,0	0.3	17.0	0.4
Nle	15.3	0.1	15.5	0.1	15.6	0.3	15.4	0.1
Pro	18.8	0.2	20.1	0.9	21.1	0.5	21.0	0.6
Asp	18,0	0.1	19.3	0.1	19.6	0.2	19.2	0.1
Glu	20.4	0.2	22.8	0.6	22.9	0.1	22.5	0.4
Phe	14.7	0.3	15,0	0.5	16.5	0.3	16.3	0.5
Tyr	15.9	0.8						
Lys	16.1	0.2	15.6	0.5	16,0	0.3	15.9	0.3
	E8AB 5M		E8AB 20M		E8AB 35M		E8AB 45 M	
AA	Mean (‰)	SD (‰)	Mean (‰)	SD (‰)	Mean (‰)	SD (‰)	Mean (‰)	SD (‰)
Ala	19.1	0.3	19.7	0.3	23.6	0.1	21.9	0.5
Gly	12.8	0.2	13.4	0.4	14.6	0.2	12.7	0.3
Thr	15.2	0.5	12,0	0.4	11.8	0.2	11.7	0.1
Ser	10.7	0.2	10.1	0.2	13,0	0.3	11.3	0.1
Val	20.8	0.6	20,0	0.5	22.4	0.2	20.9	0.1
Leu	14.4	0.1	14.9	0.2	17.4	0.2	14.5	0,0
Ile	15.6	0.2	15.9	0.1	19.2	0.5	16.1	0.1
Nle	15.2	0.1	14.3	0.1	15,0	0.2	15.6	0.1
Pro	19.1	0.1	17.5	0.6	19.9	0.0	20.1	0.3
Asp	18.1	0.3	17.8	0.2	20,0	0.1	18.4	0.1
Glu	20.9	0.1	18.4	0.1	22.3	0.2	20.8	0.2
Phe	15.3	0.2	15.3	0.2	15.3	0.2	15.3	0.2
Tyr								
Lys	14.5	0.2	14.5	0.2	14.5	0.2	14.5	0.2

S3 Table. Full data on δ^{15}N amino acids in sinking POM collected from sediment traps

	Mejillones Bay		Antofagasta Bay	
**AA**	**Mean (‰)**	**SD (‰)**	**Mean (‰)**	**SD (‰)**
**Ala**	25.7	0.5	21.6	0.8
**Val**	25.7	1.0	22.0	0.3
**Leu**	17.9	0.5	16.4	0.2
**Ile**	21.7	0.5	18.8	0.4
**Pro**	22.9	0.2	21.4	0.3
**Asp**	20.8	0.7	19.6	0.1
**Glu**	26.3	0.1	20.9	0.3
**Gly**	12.0	0.2	13.7	0.3
**Ser**	13.1	0.5	13.4	0.2
**Phe**	16.2	0.1	15.1	0.1
**Lys**	19.3	0.2	16.5	0.5
**Tyr**	12.9	0.3	15.1	0.3
**Thr**	8.8	0.4	11.3	0.3

S4 Table. Full data on apparent isotopic fractionation (Ɛx/Glu) values from suspended POM collected from MB and AB.

**Ɛx/Glu (‰)**	**MB 5m**	**SD**	**MB 25m**	**SD**	**MB 35m**	**SD**	**MB 45m**	**SD**
**Ala**	-0.6	0.5	-0.1	1.2	0.0	1.0	1.6	1.1
**Val**	0.2	0.3	-0.4	0.7	-0.6	0.1	-0.2	0.5
**Leu**	-7.2	0.4	-6.8	1.1	-6.5	0.7	-7.3	0.6
**Ile**	-5.1	0.2	-5.0	0.6	-5.0	0.7	-5.4	0.5
**Pro**	-2.6	0.4	-3.4	0.8	-3.2	0.3	-1.9	0.5
**Asp**	-2.7	0.3	-4.1	0.1	-4.1	1.0	-4.1	0.2
**Gly**	-10.3	1.4	-11.5	1.4	-11.6	0.9	-9.9	1.0
**Ser**	-10.8	0.6	-11.5	1.2	-11.9	0.6	-10.5	0.2
**Phe**	-8.2	0.3	-9.9	2.2	-9.7	0.5	-7.5	0.7
**Lys**	-7.0	2.5	-10.0	1.3	-9.3	1.2	-8.7	0.4
**Thr**	-6.2	1.9	-11.1	1.7	-14.8	1.6	-13.2	3.1
**Ɛx/Glu (‰)**	**AB 5m**	**SD**	**AB 25m**	**SD**	**AB 35m**	**SD**	**AB 45m**	**SD**
**Ala**	-0.2	1.2	0.0	0.9	0.6	0.8	0.6	0.3
**Val**	0.5	0.4	0.0	1.2	0.5	0.6	-0.3	0.2
**Leu**	-5.4	1.0	-5.4	1.5	-5.5	2.2	-6.3	0.3
**Ile**	-4.4	0.8	-4.2	1.3	-4.1	1.6	-5.0	0.3
**Pro**	-0.9	1.0	-1.2	1.1	-1.6	1.1	-1.0	0.3
**Asp**	-2.1	0.6	-1.7	1.2	-2.5	0.8	-2.6	0.5
**Gly**	-8.2	1.3	-6.4	1.2	-6.5	3.2	-6.8	1.3
**Ser**	-9.2	1.0	-9.2	1.5	-8.7	3.8	-9.9	0.5
**Phe**	-6.4	1.5	-4.8	2.9	-6.6	3.7	-6.5	0.7
**Lys**	-4.1	0.9	-6.5	1.7	-5.6	2.5	-6.3	0.1
**Thr**	-5.3	0.8	-7.5	2.1	-7.6	2.9	-9.2	0.3
**Ala**	-0.2	1.2	0.0	0.9	0.6	0.8	0.6	0.3

S5 Table. Spearman rank correlation p-values between physicochemical and $\delta^{15}N$ amino acid-derived parameters obtained from suspended POM collected from the water columns of MB and AB: $p \geq 0.1$ indicates a very weak or no correlation between variables.

Mejillones Bay					
	T	S	O2	pH	Chlorophyll-*a*
ΣV	0.2	0.2	0.2	0.1	0.1
TP Metazoan	0.8	0.6	0.8	0.6	0.1
TP Protozoan	0.2	0.2	0.2	0.8	0.2

Antofagasta Bay					
	T	S	O2	pH	Chlorophyll-*a*
ΣV	0.5	0.3	0.5	0.5	0.2
TP Metazoan	0.5	0.8	0.5	0.5	0.1
TP Protozoan	0.2	1	0.2	0.2	0.1

S6 Table. Analysis of Similarity (ANOSIM) between $\delta^{15}N$ Phe normalized $\delta^{15}N$ Ala and $\delta^{15}N$ Thr organic matter end members values and $\delta^{15}N$ Phe normalized $\delta^{15}N$ Ala and $\delta^{15}N$ Thr values found in suspended and sinking POM collected from MB and AB. An R value close to "1" suggests dissimilarity between groups while an R value close to "0" suggests an even distribution of high and low ranks within and between groups.

ANOSIM (R values)	Zoo	MDOM	FP	Phyto
**5-20 m MB**	0.7234	**0.3214**	0.7222	0.4866
**35-45 m MB**	0.3629	**-0.02183**	**0.1554**	0.8272
**5-20 m AB**	0.8517	0.7659	0.6971	0.2414
**35-45 m AB**	0.728	0.4325	0.4643	0.558
**ST MB**	**0.2419**	**0.03571**	**-0.2662**	0.9167
**ST AB**	0.9958	1	0.7922	0.3271
