# Supplementary material for: Origin, trophic transfer and recycling of particulate organic matter in two upwelling bays of Humboldt Current System: Insights from compound-specific isotopic compositions of amino acids
Source: PLoS One. 2025 May 14;20(5):e0306674. doi: 10.1371/journal.pone.0306674 (PMC12077699; doi:10.1371/journal.pone.0306674)
Supplement: Attachment — Submitted filename: Response to Reviewers.docx [file pone.0306674.s003.docx]

Universidad de Antofagasta

Av. Angamos 601, Antofagasta, Chile
biosrain@gmail.com
January 22, 2025

Vitor Hugo Rodrigues Paiva, Ph.D.
Academic Editor
PLOS ONE

Dear Vitor Hugo

Subject: Rebuttal to Manuscript Review Comments for “Origin, Trophic Transfer and Recycling of Particulate Organic Matter in Two Upwelling Bays of Humboldt Current System: Insights from Compound-Specific Isotopic Compositions of Amino Acids”

I would like to express my gratitude to you and the reviewers for the time and effort spent reviewing our manuscript. We greatly appreciate the constructive feedback, which has been invaluable in enhancing the quality of our work. Below, we address each of your comments and those of the reviewers in detail.

**I. Academic Editor**

1. Please ensure that your manuscript meets PLOS ONE's style requirements, including those for file naming.

Response: Done

2. In your Methods section, please provide additional information regarding the permits you obtained for the work. Please ensure you have included the full name of the authority that approved the field site access and, if no permits were required, a brief statement explaining why.

Response: Added in lines 152-154 of the revised manuscript.

3. Please note that funding information should not appear in any section or other areas of your manuscript. We will only publish funding information present in the Funding Statement section of the online submission form. Please remove any funding-related text from the manuscript.

Response: Edited and removed from the “Acknowledgments” section.

4. Please state what role the funders took in the study.

5. We note that your Data Availability Statement is currently as follows: All relevant data are within the manuscript and its Supporting Information files.

Response: We have provided all metadata used in the manuscript as "Supporting Information files."

We have included the statement, "The funders had no role in study design, data collection and analysis, decision to publish, or preparation of the manuscript," in the new cover letter.

**II. Reviewer # 1**

**General Comments**

**Calculation of trophic transfer is outdated**

Response: As suggested by Reviewer 1, we have calculated TP Metazoan and TP Protozoan. Please refer to lines 274 and 278 in the revised manuscript for details

**The grouping of source and trophic AAs is incorrect**
Response: We have corrected it. Please refer to Line 261 in the revised manuscript.

**Phe-normalized δ^15^N values of Ala and Thr are not calculated**

Response: These calculations were performed, and the results are discussed in the revised manuscript. Please refer to lines 304-310 for details.

**δ^15^N -THAA is not “weight-averaged”**

Response: In the revised manuscript, we calculated and presented the " mole percent weighted sum” of δ^15^N and δ^13^C -THAA" (Lines 295-302). These values were retained and discussed in the text, emphasizing their role as a "proxy" to estimate the bulk δ^15^N and δ^13^C of POM. The obtained values were compared with previously obtained δ^15^N PON and δ^13^C POC values in coastal and epipelagic waters of Chile.

**5) Additionally, the wording and phrasing need to be more specific, concise, and scientifically rigorous. There are instances of redundant wording, such as 'heavier δ^15^N enrichment,' and overly long sentences. The overall structure of the manuscript could also benefit from improvement.**

Response: We have addressed these concerns by improving the writing throughout the entire manuscript.

**Specific Comments**

**Abstract: When summarizing the findings of δ^15^N-NO_3_^-^, δ^15^N -Phe, ∆Tr and ∑V, consider reporting the actual numerical values instead of providing a general description that these parameters led to certain conclusions**

Response: We have included numerical values of ∑V, TP Metazoan and TP Protozoan in the abstract of the revised manuscript.

**LN14: The authors started the abstract with a general description of pelagic POM which does not serve as a strong motivation for this study since pelagic POM has been widely studied. Instead, highlighting POM processing in the productive and oxygen-deficient upwelling systems would be more compelling for readers. Consider emphasizing the uniqueness of the study area rather than the general importance of POM.**

Response: In the revised manuscript, we begin with a line highlighting the significance of upwelling bays and their role in primary production. Please refer to the abstract.

**LN16: Replace “chemical” by “characterizable”. With increasing depth, there is an increase in uncharacterizable POM in degraded organic matter, which sometimes constitutes the largest portion overall, whereas amino acids account for the largest portion in the characterizable portion.**

Response: This paragraph has been removed from the revised manuscript.

**LN21-22: The study objectives need to be more specific than simply “gaining insight”. Consider detailing objectives such as testing the differences between the two bays and/or examining the correlations between heterotrophic processing and oceanographic conditions**

Response: The main objectives were rethought and included as suggested by Reviewer #1 (Lines 86-93 of the revised manuscript).

**LN23-24: The ranges of δ^15^N -AA are neither important nor informative results and do not need to be reported in the abstract. I suggest reporting the average δ^15^N of source and trophic AAs (groupings to be revised – see LN211)**
Response: This paragraph has been removed from the revised manuscript. Additionally, we calculated and presented the average δ^15^N of source and trophic AAs in the revised manuscript (Lines 403-428).

**LN30: Remove “at” or “through”**

Response: Removed
**LN34: Replace “this” by “the northern Chilian”**

Response: Removed
**LN45: Replace “largest” by “****largest characterizable**

Response: Replaced in the revised manuscript.

**LN54: The explanation of why δ^15^N -AA reflects trophic transfers and degradation is not clear. The grouping of source and trophic AAs should be described here.**

Response: Addressed and rewritten in the revised manuscript (Lines 71-78).

**Methods: The overall structure of this section is disorganized. It should follow a logical order: sampling, processing, analysis, and calculations/statistics. Consider starting with the sampling of water samples and the deployment and collection of sediment traps, followed by their processing and analyses. Providing a summary paragraph detailing the parameters measured for water samples and sediment traps respectively would enhance clarity for readers.**

Response: We have reorganized and rewritten this section. Please refer to lines 96-324 in the revised manuscript

**LN111: Replace “three special stations were established” by “****three stations were established for stable nitrogen isotope analysis”**

Response: Replaced in the revised manuscript (Lines 138-139).

**LN115: Replace “These subsamples” by “****Water samples for δ^15^N N-NO_3_^-^ analysis”**

Response: Replaced. Line 145 in the revised manuscript.

**LN117: Remove “Additionally, … ocean floor” and insert a paragraph/section of sediment trap deployment here**

Response: Removed, and a paragraph/section on sediment trap deployment was added in the revised manuscript (Lines 156-164).

**LN143: The term “all samples” is confusing because the authors mentioned that amino acid δ^15^N was measured only in samples from three stations. Please specify which samples from which stations were included in the analysis.**
Response: Rewritten in the revised manuscript.

**LN157: The reproducibility of C and N of amino acid stable isotopes are typically different. It would be helpful to report specific reproducibility values for C and N separately.**

Response: Included in the revised manuscript. Please refer to lines 244 and 247 in the revised manuscript.

**LN178: Standards for nitrate δ^15^N measurements should be reported**

Response: Reported in the revised manuscript (Lines 224-227).

**Sect. 2.6: This section requires major revision. Firstly, the calculation of trophic transfer appears outdated, as newer equations based on d15N of Phe and Glu (for metazoan foodwebs, Chikaraishi et al., 2009) or Ala (for protozoan foodwebs, Décima et al., 2017) and improved β values and trophic enrichment factors from meta-analyses have been reported. It is advisable to recalculate trophic positions for metazoans (using Glu/Phe) and protozoans (using Ala/Phe), especially since the authors suggest that POM transfer is predominantly facilitated by zooplankton. Secondly, the authors should also calculate Phe-normalized Ala and Thr and compare their data with previously published end-members (zooplankton, fecal pellets, degraded OM, and phytoplankton). It would be beneficial to apply a Bayesian mixing model using these end-members to estimate the composition of OM. This approach would strengthen your interpretation regarding the degree of trophic transfer and microbial reworking.**

Response: We have recalculated trophic positions for metazoan and protozoan using Glu/Phe and Ala/Phe, respectively (Lines 274 and 278 in the revised manuscript). Additionally, we calculated Phe-normalized δ^15^N Ala and Thr (Lines 304-310) to compare with Phe-normalized δ^15^N Ala and Thr of organic matter end-members previously published in the literature and used in Bayesian mixing model analysis (Lines 325-330). All descriptions and discussions have been included in the revised manuscript.

**LN202: Remove the first “-”. Insert “δ^13^C and” between “the” and “δ^15^N”. δ^13^C and δ^15^N values of THAA are typically weight-averaged due to variations in the concentration of individual amino acids in POM, which affects the overall isotopic value. Authors should recalculate δ^13^C- and δ^15^N-THAA values based on amino acid concentration profiles. Additionally, THAA δ^13^C and δ^15^N may not accurately represent bulk δ^13^C and δ^15^N, especially for C. If feasible, authors should measure bulk stable isotopes separately and include these measurements in the study. Otherwise, all interpretations assuming that THAA represents “bulk” values should be removed from the manuscript.**

Response: In the revised manuscript, we calculated and presented the "mole percent weighted sum" of δ^15^N and δ^13^C -THAA (Lines 307-314). These values were retained and discussed in the text, emphasizing their role as a "proxy" to “estimate” the bulk δ^15^N and δ^13^C of POM. The obtained values were compared with previously obtained δ^15^N PON and δ^13^C POC values in coastal and epipelagic waters of Chile to enhance the consistency of our discussion and conclusions.

**LN211: Remove “Gly” and “Thr”. In more recent studies, Gly and Thr are not considered “source AAs” anymore, especially for Thr that gets depleted with trophic transfer. Most studies do not assign Gly and Thr to specific groupings**

Response: Gly and Ser were removed from the source AA grouping in the revised manuscript.

**LN224: Replace “AUO” by “AOU”**

Response: Replaced in the revised manuscript.

**LN256: Please specify “significant differences” in which parameters and between which groups or conditions.**

Response: It has been specified in the revised manuscript (Lines 312-316).

**Results:
LN312: The authors did not interpret chlorine index in the discussion. Consider either removing this section or moving it to the supplementary materials.
Table 1: Since the authors mentioned that the reproducibility of δ^13^C and δ^15^N measurements is better than 1‰, rounding the δ^13^C and δ^15^N values to integers does not provide sufficient precision. Most studies report stable isotope values to the tenth place**
Response: The chlorin index was removed from the manuscript. All isotopic data presented in graphs and tables are now shown to the tenth place in the revised manuscript.

**LN329-331: Given the wide ranges of δ^15^N -AA from 5 to 27‰ and 9 to 24‰, it's impossible for the standard deviations of the average values to be 0.1‰. Due to this variability, reporting the average values may not provide meaningful information**

Response: Corrected in the revised manuscript.

**LN331-332: This sentence is confusing – were the differences insignificant for all the AAs between the two bays? According to Table 2, there appear to be large differences (up to 3-4‰) in certain AAs between the two bays.**
Response: Sentence was removed and rewritten in the revised manuscript. Please refer to lines 403-428.

**LN334: Replace “most depleted” by “****least enriched”.**

Response: This phrase has been omitted from the revised manuscript.

**LN335: Remove “Thr” from source AAs**

Response: Removed

**LN367: Sediment trap δ^15^N-AA data should be included in Figure 3, with the legend clearly indicating which data are from water columns and which are from traps**
Response: The metadata for sediment traps δ^15^N-AA are provided separately in Table S3 of the Supporting Material. Values for derived δ^15^N-AA parameters for sinking POM are presented in Table 1 of the revised manuscript (Line 386).

**LN371: Recalculate the average after removing Gly and Thr**

Response: Average source-AA values were calculated excluding Gly and Thr, as described in line 272 in the revised manuscript.

**LN377: Please refer to the comments for Sect 2.6**

Response: This was addressed in previous responses, specifically concerning the observations in section 2.6

**LN399-401: This point should be discussed in the discussion section**

Response: Removed from revised manuscript.

**Table 2:** **Please refer to the comments for Table 1. Standard deviations should be reported alongside the means**

Response: Table 2 has been replaced by Table 1 in the revised manuscript. Values are presented as mean ± standard deviations (Line 386).

**Discussion:
Sect 4.1: The authors spent a considerable amount of discussion speculating on how the distribution of δ^15^N-NO_3_^-^ could be affected by various processes, despite lacking direct evidence from the current study. Please consider shortening this section and placing more focus on POM**

Response: This section has been rewritten and summarized in the revised manuscript (Lines 484-507).

**LN430: Please specify “other marine oxygen-depleted waters**

Response: Sentence rewritten in the revised manuscript (Line 487-490).

**LN436-438: The authors discussed how upwelling of ^15^N-enriched nitrogen and incomplete nitrate utilization may lead to the relatively high δ^15^N-NO_3_^-^ compared to the Pacific average. However, I disagree that incomplete nitrate assimilation by phytoplankton is a major mechanism contributing to the enriched values since we do not observe a large discrepancy between surface δ^15^N-NO_3_^-^ and δ^15^N -Phe (an indicator of phytoplankton biomass). The surface NO_3_^-^ is already ^15^N -enriched as a result of upwelled regenerated nitrogen before being assimilated into phytoplankton.**
Response: The paragraph has been removed and rewritten in the revised manuscript (Lines 491-502).

**LN453-454: The δ^15^N-NO_3_^-^ values in deep waters are highly variable between geographical locations, and comparison with the world average does not provide meaningful information. It would be more informative to compare these values with deep waters from adjacent locations or similar upwelling systems.**
Response: The paragraph has been rewritten in the revised manuscript (Lines 485-490).

**LN494: Start this sentence with POM values, stating that they are similar to δ^15^N-NO_3_^-^, as the focus here is on POM**

Response: The sentence has been rewritten in the revised manuscript.

**LN506-508: Please refer to comments regarding THAA and bulk δ^15^N values for**

Response: This was addressed in previous responses, specifically concerning the observations in LN202.

**LN202. Since THAA cannot accurately represent bulk organic material and the THAA measured in this study does not constitute a full suite of AAs, direct comparisons in δ^15^N should not be made**

Response: This was addressed in previous responses, specifically concerning the observations in LN202.

**LN521: The sentence starting with “Contrarily, it highlights the fact that” is confusing because it's unclear what 'it' refers to and why 'it' is considered contrary. Contrary to what?**

Response: Paragraph removed from the revised manuscript

**LN527-532: This sentence is overly lengthy.**

Response. Sentence has been rewritten **LN527: Please provide depth distributions of δ^15^N -TrAA and Phe-normalized δ^15^N -TrAA to support your statement regarding synchronous enrichment of d15N**

Response: This is illustrated in figure 4C and supplementary figure 5A.

**LN530: External hydrolysis is a hypothesis; please be cautious with your interpretation**

Response: The sentence has been rewritten in the revised manuscript (Lines 653-655).

**LN537-539: Move the sentence to LN532 before “In oxic…**

Response: The sentence has been relocated in the revised manuscript.

**Sect 4.2: Please start the section with a summary sentence or paragraph. There is a lot of information in the section. Consider breaking it into two subsections: one focusing on vertical profiles and the other on spatial heterogeneity**

Response: In the revised manuscript, the discussion section is divided as follows: 4.1. Dynamics of δ^15^N-NO_3_^-^ in Mejillones and Antofagasta Bays; 4.2. Isotopic Evidence for Marine-Derived, Locally Sourced POM; 4.3. Major Sources of Suspended and Sinking POM; 4.4. Vertical Patterns of Trophic Transfer and Resynthesis of POM; 4.5. Enhanced Protozoan Trophic Transfer and Bacterial Resynthesis of POM in Denitrifying Water Layers; 4.6. Spatial Variability in Trophic Transfer and Resynthesis of POM: The Role of Biological and Physical Factors.

**LN554: What are the “expected”? Please specify**

Response: The sentence was rewritten in the revised manuscript.

**LN558-559: A comparison between metazoan TP (based on δ^15^N -Glu) and protozoan TA (based on δ^15^N -Ala) will provide stronger support for this statement**

Response: This was addressed by calculating the trophic positions for metazoan and protozoan (see the Methods section in the revised manuscript). A reinterpretation of these results has been extensively discussed in the discussion subsection 'Vertical Patterns of Trophic Transfer and Resynthesis of POM' in the revised manuscript (Lines 590-659).

**LN567: There is no direct evidence for copepods in this study**

Response: It has been removed from the revised manuscript.

**LN574-576: Instead, state that “… were more enriched below 20 m depth compared to … at the surface”**

Response: This sentence was removed from the revised manuscript.

**LN578: Replace “enrichment of the heavier N isotopes” by “enrichment of the N isotopes”**

Response: This sentence was removed from the revised manuscript.

**LN581: Trophic transfers do not enrich δ^15^N of source AAs; your previous interpretation only mentioned degradation. Move LN584-587 to LN580 to support your conclusion**

Response: This sentence was removed from the revised manuscript.

**LN591-594: The increased ∆Tr and the depleted δ^15^N -Thr indicate that the higher ∑V could be due to more intense trophic transfers. Therefore, it cannot support the statement of higher microbial activity. Please revise your conclusion"**

Response: This paragraph was removed from the revised manuscript and rewritten based on the calculated TP values for Metazoan and Protozoan.

**LN599: What findings? Please specify. The authors should elaborate on how evidence of trophic transfers can be distinguished from microbial degradation and identify parameters that are affected by both processes synchronically. Additionally, please detail how microbial communities in OMZs differ from those in oxygenated waters. Currently, these statements are too ambiguous**

Response. This was addressed in the discussion section of the revised manuscript, incorporating TP values for Metazoan and Protozoan, as well as ΣV and δ^15^N values of Thr and Phe, and apparent isotopic fractionation (εx/Glu). Consequently, the paragraph in the section 'Enhanced Protozoan Trophic Transfer and Bacterial Resynthesis of POM in Denitrifying Water Layers' was rewritten.

**LN615: I disagree. The δ^15^N -Phe is lower in deeper waters but still higher compared to surface values, and the δ^15^N -Thr is depleted, indicating heterotrophic biomass**

Response: This paragraph has been removed from the revised manuscript**.**

**LN623: ∆Tr and ∑V do not imply respiration intensity or the amount and quality of exported material**

Response: It was reinterpreted, rewritten, and discussed based on our ΣV and δ^15^N values of Thr and Phe, particularly in discussion subsections 4.4, 4.5, and 4.6.

**LN628: This is just a hypothesis, so be cautious with your interpretation**

Response: It was addressed above (LN530).

**LN635: “Mesopelagic water columns” from where? Please specify the location.**
Response: This paragraph has been removed from the revised manuscript and rewritten.

**LN636: Are ∆Tr and ∑V changes correlated with the POC fluxes? Are the differences significant between the two bays?**

Response: The sample size ('n') for POC fluxes is too low to perform statistical analyses, such as correlation. However, TP values for Metazoan and Protozoan, along with ΣV and δ^15^N values of Thr and Phe, provide some inferences that attempt to explain the differences in POC fluxes between bays. These inferences are extensively discussed in the revised manuscript.

**LN644: The difference in δ^15^N -Thr offsets between the two bays is a significant finding and deserves an elaborated paragraph, together with d15N-Ala. It should be mentioned alongside the discussion on ∆Tr and ΣV**

Response: This has been elaborated in the discussion section (4.5. Spatial Variability in Trophic Transfer and Resynthesis of POM: The Role of Biological and Physical Factors) of the revised manuscript.

**LN648: Please specify what is “This heterogeneity”**

Response: This has been replaced by “Spatial variations” in the revised manuscript

**LN650: The wording of this sentence is awkward. Please revise**

Response: This sentence has been removed from the revised manuscript

**LN653-654: This sentence is confusing. It should clarify the specific parameters that are similar (oxygen, temperature, salinity, pH, and chlorophyll a) despite the different vertical CSIA-AA profiles between the two bays. Was chlorophyll a compared? Productivity may also be a factor to consider**

Response: This paragraph has been rewritten in the revised manuscript (Lines 706-711).

**LN659: This paragraph details how potential topography features in the two bays affect OM cycling. The authors effectively challenge the paradigm that water column heterogeneity alone affects OM cycling, which is a valid point. This should be mentioned in the abstract and conclusion. It could also be beneficial to discuss how coastal environments can differ significantly from pelagic environments in terms of physical, chemical, and biological interactions.**

Response: This has been added to the abstract in the revised manuscript.

**LN676: Please remove phrases like 'it was observed that' if you are already citing figures or references**

Response: Removed

**Conclusion: It could benefit from more detailed implications and outlook. Consider elaborating on how future studies might improve by including additional parameters, innovatively testing hypotheses, and addressing the implications for global and local-scale climate change**

Response: This was addressed in the conclusion section of the revised manuscript

**III. Reviewer #2**

**The manuscript is interesting and reports data on variables that are innovative for ecological research. The text is very long. Especially in the results section it should be shortened and simplified, modifying the figures and adding some other figure or tables to help the reader to understand which of the trends-variables-values are pivotal for the discussion. The discussion should be shortened, especially avoiding long hydrographic descriptions, and a little reorganized.**

Response: All of these observations have been addressed in the revised manuscript.

**Introduction
Lines 38-39: POM includes living organisms such as phytoplankton (see line 45), I would turn the sentence saying that inside POM a large fraction can be detrital**

Response: The sentences was rewritten in the revised manuscript (Lines 53-55) **Lines 90-91: Does it mean that the circulation previously described is inverted? Maybe the main hydrodynamics can be provided in Fig. 1 to help the reader**

Response: We do not have data on the velocity and direction fields of currents in both bays, so we cannot include them in Figure 1. We are only providing a description based on previous literature of the study area.

**Line 108: I never found the word “chlorine” for the sum of chlorophyll-a and phaeopigment, are you sure?**

Response: The chlorin data has been entirely removed from the revised manuscript

**Line 112: can you signal the special stations on Fig. 1 to help the reader? What is chlorine index? You describe the values in the results but do not explain how you calculated it**

Response: Figure 1 has been redrawn, and the figure caption has been revised to highlight the designated stations for carbon and nitrogen isotope analysis. The chlorin index has been removed from the updated manuscript.

**Line 116: not in station E2BM, can you specify the variables studied in the trap material as you did for the water?**

Response: This has been specified in the revised manuscript (Lines 162-164).

**Line 135: this is the method generally used for dissolved C, I suppose you did not filter the water and therefore you obtained, as you say, the total C. But how did you measure the particulate fraction?**
Response: Samples were not filtered to account for the total organic carbon contained in them. The POC analysis is described in lines 189-193 in the revised manuscript.

**Line 190: can you add a simple sentence related to the interpretation of ΣV values?
Paragraph 2.7 should be placed before paragraph 2.6, since it identifies the trophic AA.**

Response: The interpretation of ΣV values is provided in lines 291-293 of the revised manuscript.

**Line 224: correct AUO**

Response: This has been corrected in the revised manuscript.

**Results
Can you use the same scale for all the figures related to the same variable when comparing the two bays? Sometimes it is misleading to consider only the trends.
Line 263: in general, the trends of the variables are similar in the two bays, but some differences can be observed for instance at the surface. In the Discussion (line 669) you emphasise these differences, in contradiction with the Results. Horizontal plots for T at surface and 5 m depth (the bottom of the surface layer) could give a more detailed description of the variability of the hydrological features within each bay and help to discuss any differences you find in the two transects sampled for isotopic analyses.**
Response: This was addressed in the revised manuscript (see Fig. 8). The sentence in line 669 was removed and rewritten.

**Line 264: In Fig. 2, I can’t find the waters mentioned in the text. The AESS box doesn’t contain any observations, the other two boxes (ASSA-ASST) are not described-cited in the text. The Brunt Väisälä Frequency is not described, although it is reported in Fig. 6C in the Discussion, you should place this figure here (the figure is quite difficult to understand, I would divide the two areas to have larger plots with clear position of fronts and, if available, the direction of the currents).**

Response: These issues have been addressed and corrected in Figure 2 of the revised manuscript. The Brunt-Väisälä Frequency and oxygen inventories are described in lines 360-369. Additionally, Figure 6 has been redrawn and is now labeled as Figure 8 in the revised manuscript.

**Line 274: San Jorge Bay is Antofagasta Bay; you should cite this in the Study site paragraph**

Response: San Jorge Bay has been renamed and retained as Antofagasta Bay in the revised manuscript.

**Lines from 285: please add in the figures a small map of the stations, to help the reader to immediately find where the cited stations are located. For instance, the chlorophyll-a value of station E1BA at 20 m depth is unique, maybe because the station is placed next to the coast.**

Response: The paragraph has been shortened and simplified in the revised manuscript (Lines 346-359).

**Results, as often occurs due to the number of available data, are not easy to follow. The supplementary figures are “overcrowded”, for chlorophyll-a a horizontal plot focused on 5 m depth could help (the plot in the text and maintaining the S figure?). For nutrients, while NH_4_^+^ and NO_2_^-^ show scattered values, NO_3_^-^ and PO_4_^3-^ seem to follow quite regular trends with dept, but maybe at surface they have a distribution that is reflected by primary biomass (or hydrography?). Are there any significant relationships between nutrients and chlorophyll-a (or the chlorine index and TOC)? In that case, you can represent them as you did in Fig. S6 for environmental data, or in a Table. Focusing the description of the results on these observations would help the reader more than knowing the highest and the lowest values.**

Response: No significant correlations were found between these parameters.

**Line 329: I think this is suspended, not sinking. Also here to follow the text is difficult, can you refer briefly to table 1? North and south for the two transects are not only related to latitude, but to proximity to the coast or to the head of the bay, that is the opposite in the two bays. You use this way to describe the stations-data in the discussion for physical variables, maybe it is better to use it everywhere.
Why did you choose to report in Table1 the values related to Phe and Thr, both source AA, and not an example of trophic AA?**
Response. The entire section was rewritten in the revised manuscript (Lines 403-428). Table 1 was removed and changed by graphs.

**Line 390 (and maybe elsewhere): check the figure number, ΣV is panel C in figures 4 and 5.**

Response: The paragraph has been rewritten, and the figures have been modified in the revised manuscript.

**Fig.3 is complex, in the text you place together the description of trophic AA and of source AA, a figure with average values of the two kinds of AA would be easier to understand. In the supplementary material you can add a Table for the data.**

Response: This figure has been removed and replaced by the S2 Table in Supporting Material

**Discussion
The first part is related to NO_3_^-^, I would change the title of the paragraph.**

Response: Title added in the revised manuscript (Line 391).

**Lines 426-444: a division of the text in sub-paragraphs would help the reader. I would start with the denitrification influence on the entire water column (upwelling of water from OMZ) (lines 426-433), thus I would focus on the surface layer, where phytoplankton can have a role (433-436 + 438-444).**
Response: This was addressed in the revised manuscript. Please refer to lines 484 and 502; 660-684.

**Line 445: Until now you have compared your data with those coming from other areas, why do you say: another explanation…?**
Response: This was addressed in the revised manuscript. Please refer to lines 496-507.
**Line 450-454: please simplify the sentence and place the information at the beginning of the Discussion together with the comparisons with other areas.**
Response: This paragraph has been removed from the revised manuscript.

**Lines 460- 470: I would reduce the length of the paragraph and add the information at the beginning, where the relevance of nitrification in the entire water column is underlined**

The paragraph has been revised and condensed in the revised manuscript.

**Lines 471-493: I would strongly reduce the general description of the hydrodynamics and focus on the stratification (Brunt Vaisala may help).**
Response: This has been addressed in the revised manuscript. Please refer to lines 747-791.

**From line 494 to 520 the POM is concerned; I would start a new paragraph with a new title**

Response: This has been addressed in the revised manuscript. Please refer to line 508.

**Line 521-539 and following: why contrarily? Is “it” referred to the marine origin? Is this the place for ΣV? I would start here the new paragraph on microbial reworking and trophic transfer (try to simplify, remove lines 543-553).**
Response: This has been addressed in the revised manuscript. Please refer to lines 590-659. The paragraph that was previously located in lines 543-553 has been removed.

**Line 559: zooplanktonic means microbial? That is, heterotrophic? This would better link with the previous discussion on enzymatic activity. Are you sure that your data support the zooplanktonic presence in the area? This component is important, and it has the functions you indicated, but I’m not sure that your data strongly suggest this. ΔTr lower than 1 should mean that the consumers are not dominant. You should explain this point better.**
Response: This issue has been addressed in the revised manuscript by changing ΔTr to TP Metazoan and TP Protozoan parameters, as suggested by Reviewer #1.

**Line 564: surface layer is better than shallower**

Response: This has been removed from the revised manuscript.

**Lines 574-587: trophic transfer means also zooplanktonic reworking of POM? Does zooplankton live in the deep water column of the bays? I’m lost: at surface lower ΔTr means zooplankton and in the depth higher ΔTr means microbes?**
Response: This issue has been addressed in the revised manuscript by changing ΔTr to TP Metazoan and TP Protozoan parameters, as suggested by Reviewer #1.

**Line 587: Is it NH_4_^+^ or NO_3_^-^?**
Response: It refers to NH_4_^+^

**Line 588: POC decrease with depth is not surprising, given the strong pycnocline that maintain The OM produced at surface in the first meters**

**Line 628: the enzymatic activity is a function of the ecosystem that can be found everywhere, generally it depends on substrate availability**

**Response:** This paragraph has been omitted from the revised manuscript, and the availability of the substrate is now addressed in subsection 4.6 of the discussion.

**Line 639: is the difference significant?**

Response: This has been rewritten and extensively discussed in the revised manuscript.

**Line 643: Why does Thr behave differently?**

Response: This has been explained and discussed in the revised manuscript.

**In this latter part of the Discussion, I would avoid repetition of the results and hypotheses that cannot be confirmed by the data, and therefore Line 658 conclusion.**

Response: This has been removed from the revised manuscript.

**Lines 659-684: a summary of this information should be placed before, when you discuss vertical and horizontal trends**

Response: This has been relocated in the revised manuscript

**General Comments:**

We have carefully considered all the feedback and have made major revisions to the manuscript.

We believe these revisions have significantly strengthened the manuscript and addressed the reviewers' concerns. We hope that the revised manuscript meets the standards for publication in Plos One.

Thank you once again for your valuable feedback and consideration.

Sincerely,

Dr. Benjamín M. Srain, Associate Researcher at the Laboratory of Sedimentology and Paleoenvironments (LASPAL), A. von Humboldt Institute of Natural Sciences, Faculty of Marine Sciences and Biological Resources, University of Antofagasta, Antofagasta, Chile.
