# Supplementary material for: Origin, trophic transfer and recycling of particulate organic matter in two upwelling bays of Humboldt Current System: Insights from compound-specific isotopic compositions of amino acids
Source: PLoS One. 2025 May 14;20(5):e0306674. doi: 10.1371/journal.pone.0306674 (PMC12077699; doi:10.1371/journal.pone.0306674)
Supplement: Attachment — Submitted filename: Response_to_Reviewers_auresp_2.docx [file pone.0306674.s004.docx]

Universidad de Antofagasta

Av. Angamos 601, Antofagasta, Chile
biosrain@gmail.com
March 17, 2025

Vitor Hugo Rodrigues Paiva, Ph.D.
Academic Editor
PLOS ONE

Dear Vitor Hugo

Subject: Rebuttal to Manuscript Review Comments for “Origin, Trophic Transfer and Recycling of Particulate Organic Matter in Two Upwelling Bays of Humboldt Current System: Insights from Compound-Specific Isotopic Compositions of Amino Acids”

I would like to express my gratitude to you and the reviewers for the time and effort spent reviewing our manuscript. We greatly appreciate the constructive feedback, which has been invaluable in enhancing the quality of our work. Below, we address each of your comments and those of the reviewers in detail.

**I. Academic Editor**

**Please review your reference list to ensure that it is complete and correct. If you have cited papers that have been retracted, please include the rationale for doing so in the manuscript text or remove these references and replace them with relevant current references. Any changes to the reference list should be mentioned in the rebuttal letter that accompanies your revised manuscript. If you need to cite a retracted article, indicate the article’s retracted status in the References list and also include a citation and full reference for the retraction notice.**

Response: The reference list has been corrected and completed in the revised manuscript, with no retracted papers cited. Four references have been removed from the list (see lines 81-84 in the revised manuscript with track changes).

**II. Reviewer # 3**

**1. Lines 22-29. The presentation of the abstract is still somewhat vague. The authors first presented the overall characteristics of POM sources and isotopic differences, and finally merged all influencing factors together. Authors should explain each result separately, such as the reasons for the differences in POM sources and isotope values.**

Response: The abstract has been rewritten and reorganized in accordance with the reviewer' concerns.

**2. Line 394. The presentation of the "P" in statistical analysis should be italicized. Please unify the entire text.**

Response: The letter "p" in the statistical analysis has been italicized throughout the entire manuscript.

**3. Lines 662-663. N* has already been defined in the previous text, there is no need to repeat it here.**

Response: This has been corrected in the revised manuscript.

**General Comments:**

We have carefully considered all the feedback and have made minor revisions to the manuscript.

We believe these revisions have significantly strengthened the manuscript and addressed the reviewers' concerns. We hope that the revised manuscript meets the standards for publication in Plos One.

Thank you once again for your valuable feedback and consideration.

Sincerely,

Dr. Benjamín M. Srain, Associate Researcher at the Laboratory of Sedimentology and Paleoenvironments (LASPAL), A. von Humboldt Institute of Natural Sciences, Faculty of Marine Sciences and Biological Resources, University of Antofagasta, Antofagasta, Chile.
